# An Improved Spectral Subtraction Method for Eliminating Additive Noise in Condition Monitoring System Using Fiber Bragg Grating Sensors

**DOI:** 10.3390/s24020443

**Published:** 2024-01-11

**Authors:** Qi Liu, Yongchao Yu, Boon Siew Han, Wei Zhou

**Affiliations:** 1 Schaeffler Hub for Advanced Research at NTU, 61 Nanyang Dr, ABN-B1b-11, Singapore 637460, Singapore; qi.liu@ntu.edu.sg (Q.L.); yongchao.yu@ntu.edu.sg (Y.Y.); hanbon@schaeffler.com (B.S.H.); 2 School of Mechanical and Aerospace Engineering, Nanyang Technological University, 50 Nanyang Avenue, Singapore 639798, Singapore

**Keywords:** condition monitoring, signal processing, spectral subtraction, additive noise, fiber Bragg grating sensor

## Abstract

The additive noise in the condition monitoring system using fiber Bragg grating (FBG) sensors, including white Gaussian noise and multifrequency interference, has a significantly negative influence on the fault diagnosis of rotating machinery. Spectral subtraction (SS) is an effective method for handling white Gaussian noise. However, the SS method exhibits poor performance in eliminating multifrequency interference because estimating the noise spectrum accurately is difficult, and it significantly weakens the useful information components in measured signals. In this study, an improved spectral subtraction (ISS) method is proposed to enhance its denoising performance. In the ISS method, a reference noise signal measured by the same sensing system without working loads is considered the estimated noise, the same sliding window is used to divide the power spectrums of the measured and reference noise signals into multiple frequency bands, and the formula of spectral subtraction in the standard SS method is modified. A simulation analysis and an experiment are executed by using simulated signals and establishing a vibration test rig based on the FBG sensor, respectively. The statistical results demonstrate the effectiveness and feasibility of the ISS method in simultaneously eliminating white Gaussian noise and multifrequency interference while well maintaining the useful information components.

## 1. Introduction

Rotating machinery (e.g., motor and gearbox) has been widely used in many industrial applications and processes. However, faults frequently occur on rotating machinery because of regular wear and tear, adverse working conditions, overloads, and unexpected events [1,2,3,4]. To improve the availability, reliability, and safety of rotating machinery, it is necessary to develop a condition monitoring (CM) system to diagnose incipient faults and prevent sudden failures.

In the CM system, acoustic emission (AE) or vibration signals that can reflect the fault characteristics of rotating machinery are measured by various sensors (e.g., AE sensors [5], piezoelectric sensors [6], and fiber optic sensors [7]). Due to the advantages of flexibility, corrosion resistance, immunity to electromagnetic interference, small size, and light weight, the fiber optic sensors, which can be divided into three categories including point, quasidistributed, and distributed sensors, have attracted more attention [8,9,10]. The fiber Bragg grating (FBG) sensor is a common point sensor, and multiple FBGs can be inscribed into a single optical fiber to act as the quasidistributed sensor. For the distributed sensor, there are mainly two techniques including optical frequency domain reflectometry (OFDR) and optical time domain reflectometry (OTDR). The OFDR with a high spatial resolution and the OTDR with a low spatial resolution can realize continuous vibration measurement over a long distance and a wide range, whereas they provide low local position accuracy and require expensive interrogators. Compared with the OFDR and OTDR, the FBG sensor has the advantages of high sensitivity to high-frequency vibrations, high accuracy for monitoring vibrations at fixed points, and cost-efficient interrogators [11]. Thus, the FBG sensor is more suitable for monitoring crucial components of rotating machinery and has been widely applied to vibration measurement of rotating machinery [12,13,14]. However, the weak fault component in the complex measured signal may not be accurately distinguished because the measured signal is corrupted by a large amount of additive noise in the CM system using FBG sensors, which indicates that it is difficult to extract the useful and meaningful fault information.

The additive noise, such as white Gaussian noise and multifrequency interference, is intrinsic to the sensing system due to its inappropriate design [15]. To reduce the negative effects of the additive noise, various denoising methods have been increasingly developed. Common denoising methods include singular value decomposition (SVD) [16], wavelet transform (WT) [17], empirical mode decomposition (EMD) [18], and two variants (i.e., ensemble EMD [19] and complete ensemble EMD [20]), variational mode decomposition (VMD) [21], finite impulse response (FIR) filter [22], infinite impulse response (IIR) filter [23], Kalman filter [24], blind source separation (BSS) [25], and least mean square (LMS) algorithm [26]. Although the above denoising methods have been effectively applied to different real-world cases, the useful information components submerged in the strong additive noise will be weakened or destroyed to some extent. Moreover, these denoising methods are mainly used to eliminate a specific type of noise or interference, which indicates that they cannot be used to simultaneously eliminate white Gaussian noise and multifrequency interference.

The spectral subtraction (SS) method is simple and efficient in dealing with the additive noise [27]. Alonso et al. [28] successfully applied the noise suppression algorithm using the SS method to semi-automated segmentation of anuran calls. Kompella et al. [29] employed the SS method based on wavelet coefficients to reduce the influence of the noise and eliminate prefault components in the stator current. Considering that the standard SS method may introduce new noise due to the inaccurate noise estimation and nonlinear processing of negative values, researchers have investigated various improvements.

To handle different noise in an unsteady environment, a nonlinear SS method using an over-subtraction factor of nonlinear variation was developed [30]. Dahlan et al. [31] modified the nonlinear SS method by using Tsallis statistics and combined it with an unbiased minimum mean square error estimator for denoising speech. Furthermore, a multiband spectral subtraction (MBSS) method, in which spectral over-subtraction in multiple sub-bands was adopted, was developed to enhance speech signals corrupted by colored noise [32]. Peeters et al. [33,34] applied the MBSS and wavelet denoising methods to reduce the amount of noise in vibration signals when diagnosing vibration-based bearing faults. However, in the MBSS method, the noise may be inaccurately estimated, and the fixed spectral floor factor cannot maximize the reduction in the residual noise under different noise. For this problem, Tong et al. [35] introduced an adaptive noise estimation method and the corresponding spectral floor factors under different signal-to-noise ratios (SNRs).

Additionally, the standard SS method was modified by combining with different strategies. Miyazaki et al. [36] developed a musical-noise-free blind speech extraction method by applying multiple iterative SS to each channel. An improved frame iterative SS method in the short-time modulation domain was presented by Li et al. [37] to enhance the residual music noise in the low SNR scenarios, in which the noise subtraction was executed to deal with the signal for each frame in the short time modulation domain by using the interframe correlation. To identify surface intrusion event signals measured by an FBG array, Xin et al. [38] combined the SS method with the root mean square of power spectral density. Ozawa et al. [39] designed a novel sound source separation method based on two-dimensional fast Fourier transform (FFT), in which the noise spectrum was obtained by a deep neural network and subtracted from the measured signal. Furthermore, they replaced the deep neural network with a formula obtained by mathematical derivation to estimate the noise spectrum. Balaji et al. [40] proposed a modified SS method by adding two additional parameters (i.e., the over-subtraction and spectral floor factors), using the spectral subtraction independently in each sub-band, and combining with the Wiener filter.

Although the principle of the SS method makes it possible to simultaneously eliminate white Gaussian noise and multifrequency interference, the SS method and its variants are mainly applied to the removal of white Gaussian noise at present. This is because their denoising capability is significantly affected by the noise estimation. In the SS method and its variants, the noise spectrum is estimated as accurately as possible using the over-subtraction factor or machine learning methods. Unfortunately, the strong additive noise cannot be effectively removed because the deviation between the estimated and actual noise spectrums may be large. In particular, the SS method and its variants lack the ability to remove the multifrequency interference generated by the sensing system due to the difficulty of estimating the corresponding noise spectrum. Another drawback of the SS method and its variants is that the useful information components submerged in the strong additive noise will also be weakened or destroyed to some extent.

The main purpose of this study is to propose an improved spectral subtraction (ISS) method for effectively eliminating the additive noise in the CM system using FBG sensors. In the ISS method, three modifications are introduced into the standard SS method. The denoising performance of the ISS method is evaluated by conducting a simulation analysis based on simulated signals and an experiment based on a vibration test rig using the FBG sensor. Compared with the SS method and its variants, the ISS method has four significant improvements as follows:

- The estimated noise is replaced with a reference noise signal measured by the same sensing system without working loads, which can effectively reduce the negative influence of the inaccurate noise estimation on the denoising performance;
- Considering the difference between the noise in the measured signal and the reference noise signal, the power spectrums of the two signals are divided into multiple frequency bands by using the same sliding window, and the formula of spectral subtraction in the standard SS method is modified by using the maximum amplitude of the power spectrum in each frequency band of the reference noise signal;
- The white Gaussian noise and multifrequency interference can be simultaneously eliminated by the ISS method;
- The ISS method hardly weakens or destroys the useful information components submerged in the strong additive noise, which leads to that the useful information components can be well maintained.

The rest of this paper is organized as follows. Section 2 provides the methodology in detail. Section 3 analyzes the denoising performance of the ISS method by using simulated signals. Section 4 validates the denoising performance of the ISS method by using the vibration signals measured by the FBG sensor. Finally, Section 5 gives conclusions and suggestions for further work.

## 2. Methodology

### 2.1. FBG Sensor

An FBG sensor can reflect the spectrum of incident light with a specific wavelength [41]. According to the coupled-mode theory of the optical fiber, the Bragg wavelength $\lambda_{B}$ is given as follows:

(1) $$\lambda_{B}=2n_{neff}\Lambda$$

where $n_{neff}$ denotes the effective refractive index, and Λ denotes the grating period.

The shift of the Bragg wavelength of the FBG sensor, which is induced by the periodical variation in the refractive index, is sensitive to the change in strain and temperature. Thus, the shift of the Bragg wavelength $\Delta \lambda_{B}$ caused by the strain ε and the temperature ΔT is expressed as follows [42]:

(2) $$\Delta \lambda_{B}=\lambda_{B}\left[\varepsilon \left(1-P_{e}\right)+\left(\alpha +\xi \right)\Delta T\right]$$

where $P_{e}$ denotes the effective photo-elastic coefficient, α denotes the thermal expansion coefficient, and ξ denotes the thermal-optic coefficient.

Since all experiments in this study are completed at normal room temperature and in a short time, the shift of the Bragg wavelength $\Delta \lambda_{B}$ is only caused by the strain ε, which is expressed as follows:

(3) $$\Delta \lambda_{B}=\lambda_{B}\left[\varepsilon \left(1-P_{e}\right)\right]$$

### 2.2. Spectral Subtraction

**Figure 1:**
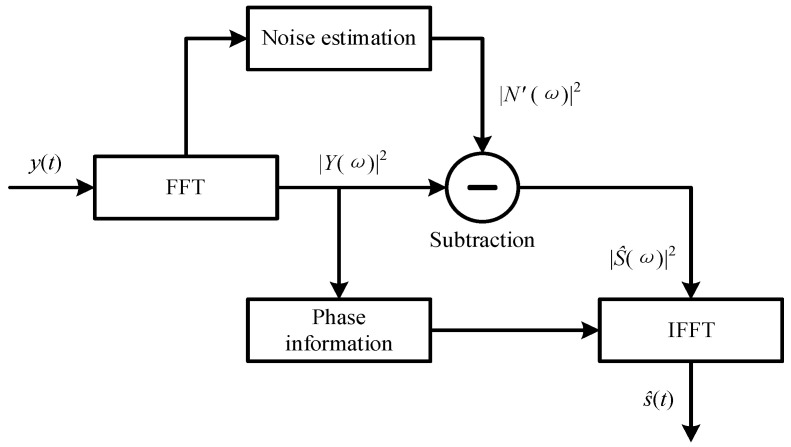
Flowchart of the spectral subtraction method.

The spectral subtraction (SS) method was developed for eliminating the additive noise. The flowchart of the SS method is provided by Figure 1, and its description is given below.

Suppose $s\left(t\right)$ denotes an original signal, and $n\left(t\right)$ denotes an additive noise. A measured noisy signal $y\left(t\right)$ is given as follows:

(4) $$y\left(t\right)=s\left(t\right)+n\left(t\right)$$

Then, the power spectrum of $y\left(t\right)$ is obtained by the FFT method and given by Equation (5).

(5) $${\left|Y\left(\omega \right)\right|}^{2}={\left|S\left(\omega \right)\right|}^{2}+{\left|N\left(\omega \right)\right|}^{2}$$

where $Y\left(\omega \right)$, $S\left(\omega \right)$, and $N\left(\omega \right)$ are complex numbers at each frequency, and their amplitudes are $\left|Y\left(\omega \right)\right|$, $\left|S\left(\omega \right)\right|$, and $\left|N\left(\omega \right)\right|$. The phase of $Y\left(\omega \right)$ is represented as $\theta \left(\omega \right)$.

The power spectrum of the denoised signal ${\left|\hat{S}\left(\omega \right)\right|}^{2}$ is obtained by subtracting the power spectrum of the estimated noise ${\left|N^{\prime }\left(\omega \right)\right|}^{2}$ from the power spectrum of the measured noisy signal ${\left|Y\left(\omega \right)\right|}^{2}$, which is formulated as follows:

(6) $${\left|\hat{S}\left(\omega \right)\right|}^{2}={\left|Y\left(\omega \right)\right|}^{2}-{\left|N^{\prime }\left(\omega \right)\right|}^{2}$$

Finally, considering that the phase of the denoised signal is approximately the same as the phase of the measured noisy signal, the denoised signal $\hat{s}\left(t\right)$ is achieved by the inverse fast Fourier transform (IFFT) method, which is formulated as follows:

(7) $$\hat{s}\left(t\right)=\mathrm{IFFT}\left[\left|\hat{S}\left(\omega \right)\right|\cdot e^{j\theta \left(\omega \right)}\right]$$

### 2.3. Improved Spectral Subtraction Method for Eliminating the Additive Noise

**Figure 2:**
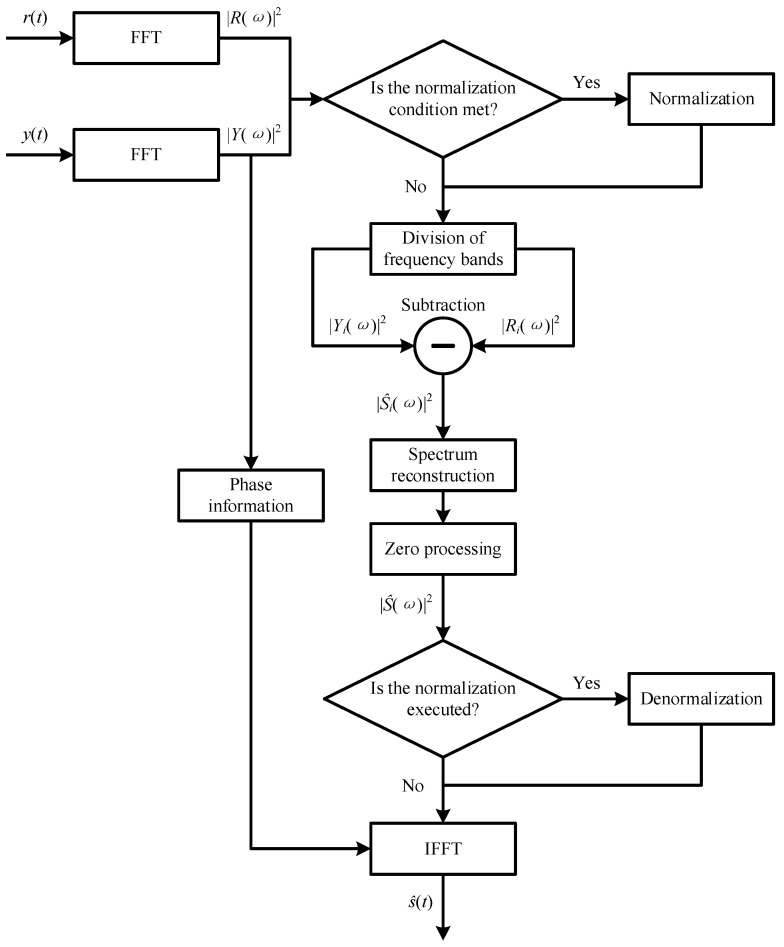
Flowchart of the improved spectral subtraction method.

To effectively eliminate the additive noise in the CM system using FBG sensors, an improved spectral subtraction (ISS) method is proposed by introducing three modifications into the standard SS method in this work, and its flowchart is provided in Figure 2.

For the first modification, since estimating the noise spectrum accurately is difficult, a reference noise signal measured by the same sensing system without working loads is considered the estimated noise in this work. The reference noise signal is represented as $r\left(t\right)$, and its power spectrum is represented as ${\left|R\left(\omega \right)\right|}^{2}$.

Considering the difference between the noise in the measured noisy signal and the reference noise signal, the other two modifications are provided as follows:

- The same sliding window is used to divide the power spectrums of the measured noisy and reference noise signals into multiple frequency bands, and there is an overlap between the power spectrums in two neighboring frequency bands. An appropriate window length should be determined by analyzing the difference between the power spectrums of the measured noisy and reference noise signals. To eliminate the additive noise more effectively, when the useful information component in the measured noisy signal is different from the additive noise and the amplitude of the useful information component in the power spectrum of the measured noisy signal is smaller than the maximum amplitude of the power spectrum of the additive noise, the normalization processing can be executed before dividing the two power spectrums.
- Equation (6) is modified into Equation (8). As shown in Equation (8), the maximum amplitude of the power spectrum in each frequency band of the reference noise signal is subtracted from the amplitude of the power spectrum in each frequency band of the measured noisy signal.

(8) $${\left|{\hat{S}}_{i}\left(\omega \right)\right|}^{2}={\left|Y_{i}\left(\omega \right)\right|}^{2}-\max\left({\left|R_{i}\left(\omega \right)\right|}^{2}\right)$$

where *i* = 1, 2, …, *Num*, here, *Num* is the number of frequency bands.

After spectral subtraction is conducted, the amplitude of the power spectrum in each frequency band is set to zero when it is negative. Meanwhile, the mean values of the overlapping components are calculated. Since a small mean value indicates less noise in the overlapping component, the overlapping component with a smaller mean value is selected. Then, the reconstructed power spectrum can be obtained.

There may be several small independent peaks at random positions in the reconstructed power spectrum because the negative value obtained by spectral subtraction is set to zero. Thus, the peak value that is less than the mean value of all peaks is set to zero, and then the reconstructed power spectrum is updated, which is represented as ${\left|\hat{S}\left(\omega \right)\right|}^{2}$. Additionally, if the normalization processing is executed, the denormalization processing should be executed in terms of the normalization processing before IFFT to recover the amplitude information of the measured noisy signal.

## 3. Simulation Analysis

In this section, the performance of the ISS method in eliminating the additive noise including white Gaussian noise and multifrequency interference is quantitatively evaluated by using simulated signals.

### 3.1. Performance Metrics

Four performance metrics [43,44], which include SNR, mean square error (MSE), percentage root mean square difference (PRD), and normalized correlation coefficient (NCC), are used to assess the denoising performance of the ISS method.

SNR is used to verify the quality of the denoised signal. The higher the SNR value, the better the denoising performance. The definition of SNR is expressed as follows:

(9) $$\mathrm{SNR}=10\log_{10}\left(\frac{\sum_{i=1}^{N}s_{original}^{2}\left(i\right)}{\sum_{i=1}^{N}{\left[s_{denoise}\left(i\right)-s_{original}\left(i\right)\right]}^{2}}\right)$$

where $s_{original}$ is the original signal, $s_{denoise}$ is the denoised signal, and *N* is the sampling length of the signal.

MSE is used to reflect the extent to which the denoised signal deviates from the original signal. The smaller the MSE value, the smaller the difference between the two signals. The formula of MSE is given as follows:

(10) $$\mathrm{MSE}=\frac{1}{N}\sum_{i=1}^{N}{\left[s_{denoise}\left(i\right)-s_{original}\left(i\right)\right]}^{2}$$

PRD, which denotes the reconstruction accuracy by a point-to-point correlation with the original signal, is used to quantify the distortion of the denoised signal. The smaller the PRD value, the more similar the denoised signal is to the original signal. This performance metric is characterized as follows:

(11) $$\mathrm{PRD}=\sqrt{\frac{\sum_{i=1}^{N}{\left[s_{denoise}\left(i\right)-s_{original}\left(i\right)\right]}^{2}}{\sum_{i=1}^{N}s_{original}^{2}\left(i\right)}}\times 100$$

NCC is used to evaluate the correlation between the denoised signal and the original signal. The closer the NCC value is to 1, the higher the correlation between the two signals. The expression of NCC is given as follows:

(12) $$\mathrm{NCC}=\frac{\sum_{i=1}^{N}s_{original}\left(i\right)s_{denoise}\left(i\right)}{\sqrt{\sum_{i=1}^{N}s_{original}^{2}\left(i\right)\sum_{i=1}^{N}s_{denoise}^{2}\left(i\right)}}$$

### 3.2. Performance Analysis

In the real-world CM system using FBG sensors, the measured vibration signals are usually composed of multiple useful vibration components and strong additive noise including white Gaussian noise and multifrequency interference generated by the sensing system. To analyze the denoising performance of the ISS method, a simulated noisy signal $y\left(t\right)$, which consists of a multifrequency sinusoidal signal (i.e., original signal) $s\left(t\right)$, a multifrequency interference $n_{1}\left(t\right)$, and a white Gaussian noise $n_{2}\left(t\right)$, is used and presented as follows:

(13) $$y\left(t\right)=s\left(t\right)+n_{1}\left(t\right)+n_{2}\left(t\right)$$

where the formulae of $s\left(t\right)$ and $n_{1}\left(t\right)$ are given as follows:

(14) $$s\left(t\right)=\sum_{i=1}^{3}A_{i}^{s}\sin\left(2\pi f_{i}^{s}t\right)$$

(15) $$n_{1}\left(t\right)=\sum_{i=1}^{5}A_{i}^{n}\sin\left(2\pi f_{i}^{n}t\right)$$

where $A_{i}^{s}$ and $f_{i}^{s}$ are the amplitude and frequency of the multifrequency sinusoidal signal, respectively. Meanwhile, $A_{i}^{n}$ and $f_{i}^{n}$ are the amplitude and frequency of the multifrequency interference, respectively. Considering that the multifrequency sinusoidal signal is used to represent the useful vibration components, $A_{1}^{s}-A_{3}^{s}$ are set to 0.65, 0.85, and 0.75, respectively, and $f_{1}^{s}-f_{3}^{s}$ are set to 300 Hz, 500 Hz, and 600 Hz, respectively. Generally, the multifrequency interference, whose frequencies are close to those of the useful vibration components and whose amplitudes are higher than those of the useful vibration components, has a significant negative influence on the analysis of the useful vibration components and is difficult to eliminate. Additionally, the multifrequency interference, which is significantly different from the useful vibration components, should also be eliminated. Thus, $A_{1}^{n}-A_{5}^{n}$ are set to 1, 0.7, 1, 0.8, and 0.3, respectively, and $f_{1}^{n}-f_{5}^{n}$ are set to 100 Hz, 350 Hz, 505 Hz, 650 Hz, and 800 Hz, respectively.

To analyze the effect of the noise intensity of the additive noise on the denoising performance of the ISS method, the noise intensity of white Gaussian noise is set from 0 dBW to 20 dBW at an interval of 5 dBW. All simulated noisy signals are sampled at a sampling frequency of 2000 Hz and a sampling length of 10,000. Then, the SNRs of the simulated noisy signals are calculated by using Equation (9), and their values are −4.937 dB, −7.385 dB, −11.410 dB, −15.871 dB, and −20.706 dB, respectively.

**Figure 3:**
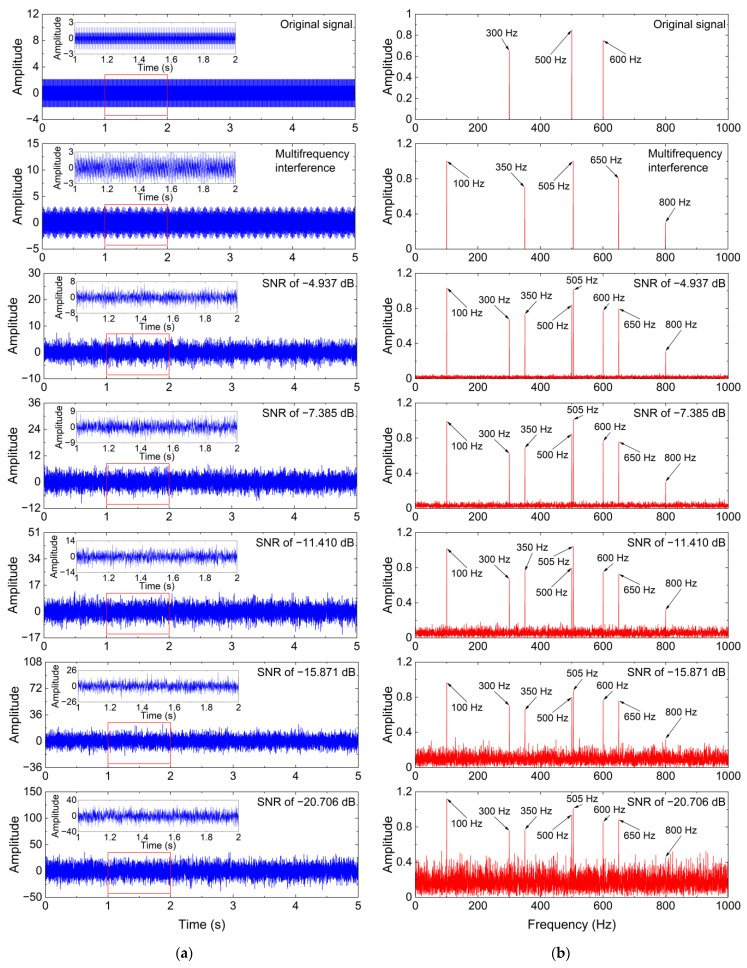
Simulated signals and their frequency spectrums. (**a**) Time-domain waveform; (**b**) Frequency spectrum.

Figure 3 shows time-domain waveforms and frequency spectrums of the multifrequency sinusoidal signal, multifrequency interference, and simulated noisy signals with different SNRs. As shown in Figure 3, the multifrequency sinusoidal signal is significantly affected by white Gaussian noise and multifrequency interference, and the negative effect of the additive noise increases with the increase in the noise intensity. Thus, the ISS method is employed to eliminate the additive noise in the simulated noisy signals with different SNRs. Meanwhile, the denoising performance of the ISS method is compared with that of the standard SS and MBSS methods. For the ISS method, the window length is set to 30, and the reference noise signals are randomly generated in terms of the additive noise. The simulation analysis is executed by using the MATLAB R2022a installed on a computer with an Intel^®^ Core™ i7-12700H CPU@2.70 GHz (Intel, Santa Clara, CA, USA), 16.00 GB of RAM (Micron Technology, Boise, ID, USA), and a Windows 11 64-bit system.

Table 1 records the performance metrics achieved by the ISS method and two comparative denoising methods for the simulated noisy signals with different SNRs. In Table 1, BSNR denotes the SNR of the simulated noisy signal, and ASNR denotes the SNR of the denoised signal.

As can be seen from Table 1, with the decrease in the BSNR value, the ASNR and NCC values obtained by the three denoising methods decrease, and the MSE and PRD values obtained by the three denoising methods increase. The phenomenon shows that the denoising performance of the three denoising methods decreases as the SNR of the simulated noisy signal decreases. However, the ISS method obtains the largest ASNR and NCC values and the smallest MSE and PRD values regardless of the SNR of the simulated noisy signal, which indicates that the denoising performance of the ISS method is significantly superior to that of the SS and MBSS methods. Meanwhile, in terms of the performance metrics achieved by the three denoising methods, there are significant differences between the denoised signals obtained by the SS and MBSS methods and the original signal, whereas the denoised signals obtained by the ISS method are significantly similar to the original signal. In other words, different from the SS and MBSS methods, the ISS method can not only effectively remove white Gaussian noise and multifrequency interference from the simulated noisy signals but also well maintain the useful information components.

In summary, the denoising performance of the SS method is significantly improved by introducing three modifications, and the ISS method has a strong ability to eliminate the additive noise including white Gaussian noise and multifrequency interference.

## 4. Experimental Validation

In this section, the effectiveness and feasibility of the ISS method in real-world applications are validated by establishing a vibration test rig based on the FBG sensor.

### 4.1. Experimental Setup

**Figure 4:**
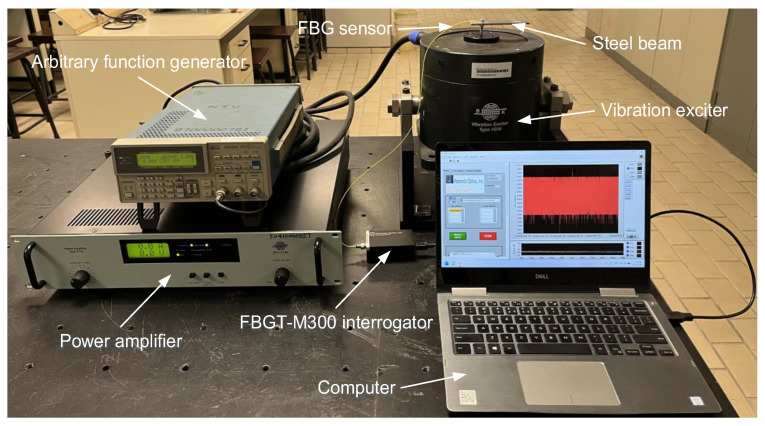
Vibration test rig based on the FBG sensor.

The vibration test rig based on the FBG sensor is established by using a steel beam, a single-mode FBG sensor (OSC1100, TongWei Technology Co., Ltd., Beijing, China), an interrogator (FBGT-M300, Redondo Optics, Inc., Redondo Beach, CA, USA), a vibration exciter (Type 4808, Brüel & Kjær, Nærum, Denmark), an arbitrary function generator (AFG320, Tektronix, Beaverton, OR, USA), a power amplifier (Type 2719, Brüel & Kjær, Nærum, Denmark), and a computer. The physical diagram of the vibration test rig is shown in Figure 4.

The beam with dimensions of 100 mm × 20 mm × 2 mm is fabricated by 304 stainless steel. For the FBG sensor, it is fabricated by a 248 nm KrF excimer laser using the phase mask technique, the Bragg wavelength is 1549.94 mm, the grating length is 10 mm, the full-width at half maximum (FWHM) is 0.23 nm, the reflectivity is 92.23%, the side-lobe suppression ratio (SLSR) is 24 dB, the coating type is acrylate, the grating area is not coated, and the operational temperature ranges from −40 °C to 120 °C. The FBGT-M300 interrogator with dimensions of 78 mm × 47 mm × 27 mm can use an optical channel to simultaneously interrogate three FBG sensors at a sampling frequency of 20 kHz. The AFG320 arbitrary function generator can output a sinusoidal signal with a frequency range of 0.01 Hz to 16 MHz and an amplitude range of 50 mV to 10 V. The Type 4808 vibration exciter has a frequency range of 5 Hz to 10 kHz, a maximum displacement of 12.7 mm, and a force rating of 112 N. The Type 2719 power amplifier has a frequency range of DC to 100 kHz and is designed specifically to drive the Type 4808 vibration exciter.

As shown in Figure 4, the steel beam is installed on the vibration exciter by a screw. The vibration exciter is connected to the arbitrary function generator through the power amplifier to excite vibration signals with different excitation frequencies. To ensure that the vibration signal on the steel beam can be effectively transmitted to the FBG sensor, the surface of the steel beam is polished with a grinder to make it as flat as possible and cleaned with alcohol. Then, the FBG sensor is connected to the FBGT-M300 interrogator by using the FC/APC optical connector and pasted on the steel beam with UV shadowless glue (Ergo 8500, Kissling AG, Wetzikon, Switzerland) to collect the vibration signal. The UV shadowless glue with a viscosity of 1200 mpas and an operational temperature range of −30 °C to 100 °C has the advantages of antipeeling, antistretching, antivibration, antidrop, no corrosion to items, and fast curing time, and it has a strong ability to bond metal, plastic, and glass parts in any combination. Aiming at recording and displaying the vibration signal and providing power to the FBGT-M300 interrogator, the computer is connected to the FBGT-M300 interrogator by using USB data communication.

**Figure 5:**
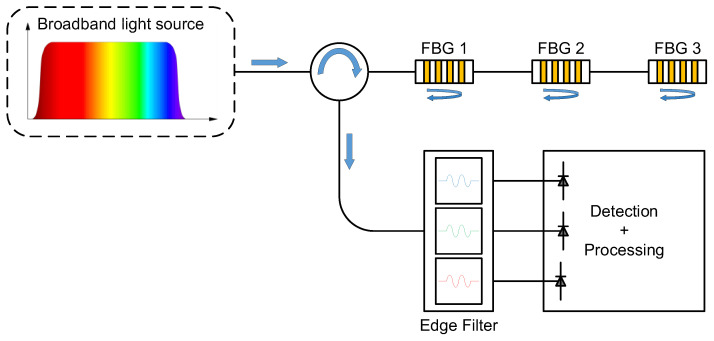
Measurement principle of FBGT-M300 interrogator based on the edge filtering method.

The measurement principle of the FBGT-M300 interrogator, as shown in Figure 5, is based on the edge filtering method that allows the interrogator to work at a high frequency. A light is injected into the optical fiber by a broadband light source. Then, the passive demodulation technique based on optical edge filters is used in the FBGT-M300 interrogator, which means that the optical signal (i.e., the shift of the Bragg wavelength) from each FBG sensor is converted into an electrical signal at each photodiode by the optical properties of the optical edge filter after the reflected light is sent into three optical edge filters centered on the Bragg wavelength of each FBG sensor. In other words, the light intensity is measured and a voltage proportional to the shift of the Bragg wavelength is provided by a photodiode at the output of each optical edge filter associated with each FBG sensor. The Bragg wavelength varies linearly with an external strain due to the use of the optical edge filter, and it is visualized by the output voltage. However, the bandwidth and the number of filters limit the number of FBG sensors interrogated simultaneously. In the FBGT-M300 interrogator, all the functionalities of the key passive and active optoelectronics components are monolithically integrated into a microchip. Meanwhile, a microprocessor controller, which is mounted on a complementary metal oxide semiconductor (CMOS)-PC board equipped with conventional flash memory, data storage, and USB data transmission elements, is used to acquire and process the converted electrical signal (i.e., the output voltage) carrying the information from each FBG sensor.

**Figure 6:**
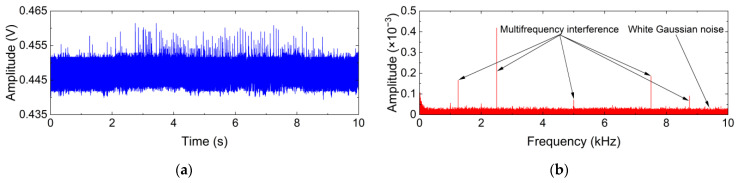
Additive noise generated by the FBGT-M300 interrogator and its frequency spectrum. (**a**) Time-domain waveform; (**b**) Frequency spectrum.

The additive noise generated by the FBGT-M300 interrogator, which includes white Gaussian noise and multifrequency interference, is shown in Figure 6. Since the dynamic range is limited to 10 nm, the weak signal will lead to a low variation output voltage that is equivalent to the noise level, which indicates that the weak useful information component is submerged in the strong additive noise.

### 4.2. Performance Analysis

The vibration of the steel beam is excited by the vibration exciter, and its amplitude and frequency can be controlled by adjusting the output amplitude and frequency of the arbitrary function generator. For the vibration of the steel beam excited in this experiment, it is set to the sinusoidal signal, its amplitude is set to 1 V, and its frequency is set from 800 Hz to 1050 Hz at an interval of 50 Hz. The vibration signals with different excitation frequencies on the steel beam are collected by the FBG sensor at a sampling frequency of 20 kHz and a sampling length of 200,000.

**Figure 7:**
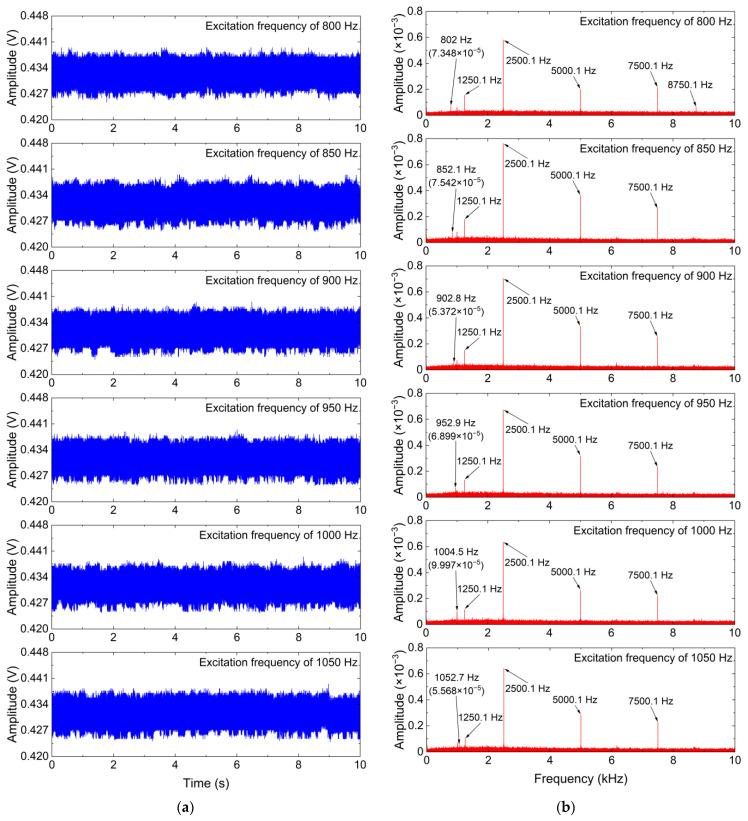
Measured vibration signals with different excitation frequencies and their frequency spectrums. (**a**) Time-domain waveform; (**b**) Frequency spectrum.

Figure 7 shows time-domain waveforms and frequency spectrums of measured vibration signals with different excitation frequencies. As shown in Figure 7, the frequencies of the useful vibration components are 802 Hz, 852.1 Hz, 902.8 Hz, 952.9 Hz, 1004.5 Hz, and 1052.7 Hz, respectively, and the useful vibration components are weak and submerged in the strong additive noise. Thus, the ISS method is employed to eliminate the additive noise in the measured vibration signals with different excitation frequencies, and its denoising performance is also compared with that of the standard SS and MBSS methods. For the ISS method, the window length is set to 30. The denoising results for the measured vibration signals with different excitation frequencies are obtained by the ISS method and two comparative denoising methods.

To quantitatively evaluate the denoising performance of the ISS method, the original signals are estimated in terms of the output of the vibration exciter, and the performance metrics achieved by the ISS method and two comparative denoising methods are provided in Table 2. As shown in Table 2, the ISS method exhibits the best denoising performance for all the measured vibration signals because it obtains the smallest MSE and PRD values and the largest ASNR and NCC values. Meanwhile, the performance metrics achieved by the ISS method are far different from those of the SS and MBSS methods, which indicates that the denoising performance of the ISS method is significantly better than that of the two comparative denoising methods.

**Figure 8:**
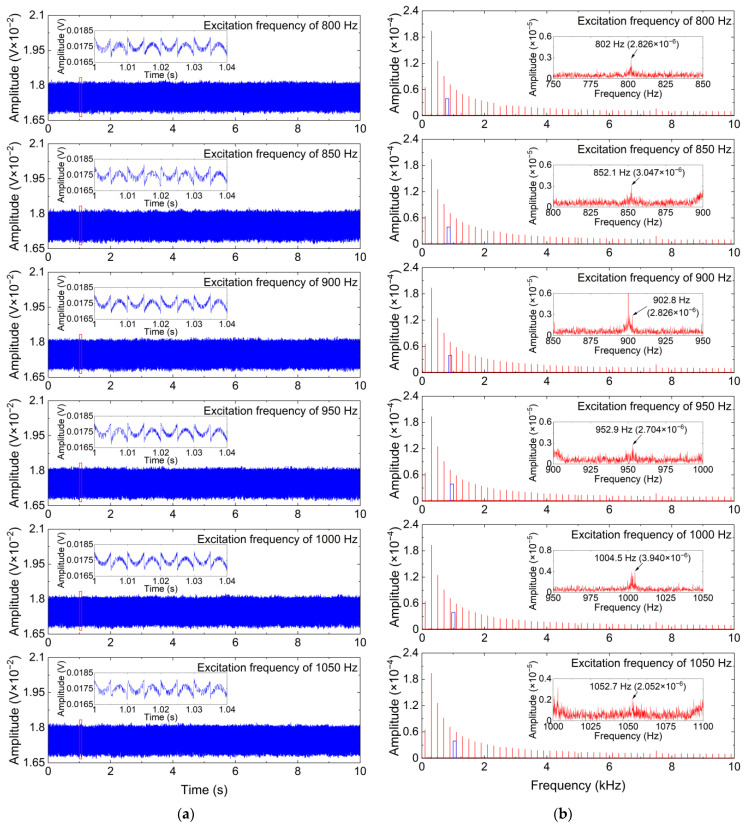
Denoising results obtained by the SS method for measured vibration signals with different excitation frequencies. (**a**) Time-domain waveform; (**b**) Frequency spectrum.

**Figure 9:**
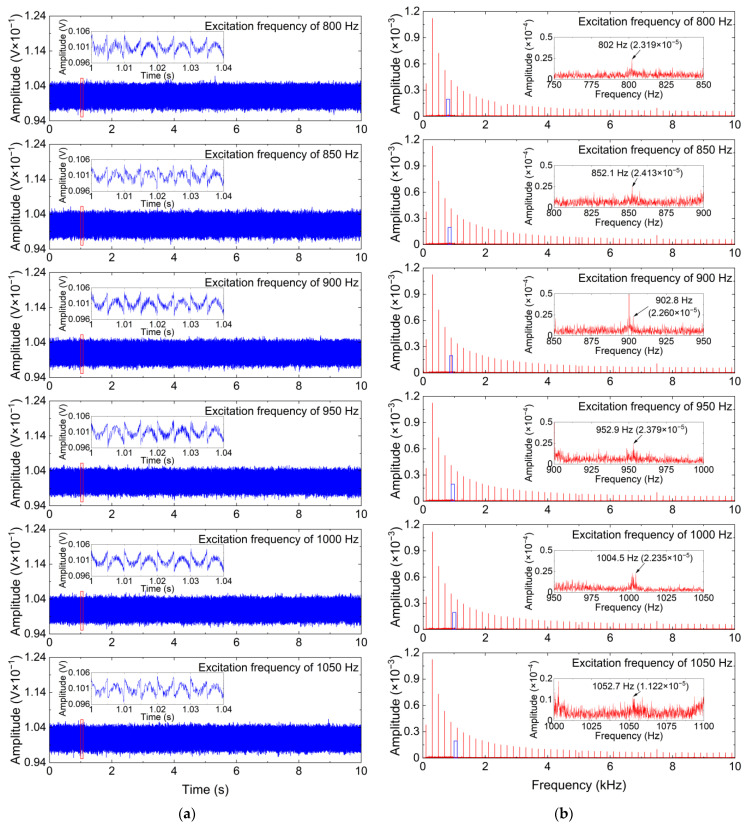
Denoising results obtained by the MBSS method for measured vibration signals with different excitation frequencies. (**a**) Time-domain waveform; (**b**) Frequency spectrum.

**Figure 10:**
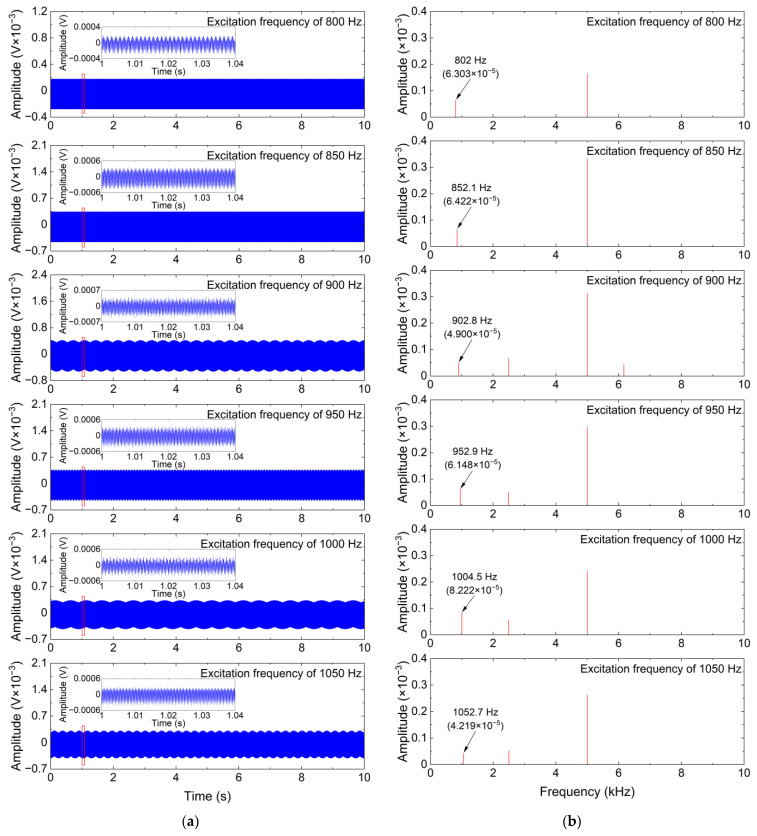
Denoising results obtained by the ISS method for measured vibration signals with different excitation frequencies. (**a**) Time-domain waveform; (**b**) Frequency spectrum.

To further clearly analyze the denoising performance of the ISS method, time-domain waveforms and frequency spectrums of the denoising results obtained by the ISS method and two comparative denoising methods for the measured vibration signals with different excitation frequencies are shown in Figure 8, Figure 9 and Figure 10.

As can be seen from Figure 8 and Figure 9, the SS and MBSS methods not only fail to eliminate the additive noise in the measured vibration signals but also introduce new additive noise into the measured vibration signals, which indicates that the two methods cannot undertake the challenge of eliminating the additive noise in the CM system using FBG sensors. The reason for the failure of the SS and MBSS methods is the inaccurate noise estimation. As shown in Figure 10, the weak useful vibration components, whose frequencies are similar to the excitation frequencies, can be effectively distinguished after the additive noise is removed by the ISS method from the measured vibration signals. Although the time-domain waveforms of the denoising results obtained by the ISS method have obvious distortion due to the existence of residual interference, the weak useful vibration components are more obvious in terms of the frequency spectrums of the denoising results obtained by the ISS method, and the denoised vibration signals can be used for signal analysis or feature extraction in future work. Another impressive phenomenon is that the amplitudes of the useful vibration components are dramatically weakened by the SS and MBSS methods, whereas the amplitudes of the useful vibration components in the denoising results obtained by the ISS method are slightly smaller than that of the useful vibration components in the measured vibration signals. This phenomenon demonstrates that the ISS method is capable of simultaneously eliminating white Gaussian noise and multifrequency interference without weakening or destroying the useful vibration components as much as possible. In other words, the ISS method significantly outperforms the SS and MBSS methods in removing the additive noise from the vibration signals measured by the FBG sensor.

According to the analysis results, we can conclude that the ISS method is feasible and effective for eliminating the additive noise in the CM system using FBG sensors.

## 5. Conclusions

In this study, an improved spectral subtraction (ISS) method is proposed to effectively eliminate the additive noise in the CM system using FBG sensors. In the ISS method, three modifications are introduced into the standard SS method. To avoid the negative influence of the inaccurate noise estimation on the denoising performance of the SS method, a reference noise signal measured by the same sensing system without working loads is used to replace the estimated noise. Meanwhile, considering that the noise in the measured signal is different from the reference noise signal, the same sliding window is used to divide the power spectrums of the two signals into multiple frequency bands, and the maximum amplitude of the power spectrum in each frequency band of the reference noise signal is used to modify the formula of spectral subtraction in the standard SS method.

A simulation analysis is executed by using simulated noisy signals which consist of a multifrequency sinusoidal signal, a multifrequency interference, and white Gaussian noise with five different noise intensities. The statistical results prove the superiority of the ISS method over the SS and MBSS methods. Additionally, an experiment is conducted by establishing a vibration test rig based on the FBG sensor. The statistical results demonstrate that the ISS method is more effective than the SS and MBSS methods for eliminating the additive noise in the CM system using FBG sensors. Different from the SS and MBSS methods, the ISS method can simultaneously eliminate white Gaussian noise and multifrequency interference while well maintaining the useful information components.

According to the above analysis, we believe that the ISS method can provide the acceptable performance in eliminating the additive noise. However, there are still some limitations to overcome. Since some useful vibration components in the measured vibration signal are much weaker than the multifrequency interference, the multifrequency interference cannot be completely eliminated by the ISS method. Moreover, due to the principle of the ISS method, this denoising method cannot be used to eliminate the interference which is concentrated in a frequency band quite similar to that of the useful information component in the measured signal and whose amplitude is significantly higher than that of the useful information component in the measured signal. Thus, in future work, the ISS method combined with other denoising methods or strategies should be researched to enhance its suitability and practicability for real-world applications.

## Figures and Tables

**Table 1:** Performance metrics achieved by the ISS method and two comparative denoising methods for simulated noisy signals with different SNRs.

BSNR (dB)	Performance Metric	SS	MBSS	ISS
−4.937	ASNR (dB)	0.329	0.825	31.110
	MSE	7.914 × 10^−1^	7.061 × 10^−1^	6.612 × 10^−4^
	PRD	96.279	90.940	2.783
	NCC	0.3113	0.4268	0.9996
−7.385	ASNR (dB)	0.270	−0.593	28.279
	MSE	8.023 × 10^−1^	9.787 × 10^−1^	1.269 × 10^−3^
	PRD	96.940	107.067	3.855
	NCC	0.2456	0.3055	0.9994
−11.410	ASNR (dB)	−0.497	−3.513	24.920
	MSE	9.573 × 10^−1^	1.917	2.750 × 10^−3^
	PRD	105.890	149.856	5.676
	NCC	0.1441	0.2093	0.9990
−15.871	ASNR (dB)	−1.155	−7.187	19.773
	MSE	1.114	4.467	8.996 × 10^−3^
	PRD	114.221	228.751	10.265
	NCC	0.0892	0.1378	0.9965
−20.706	ASNR (dB)	−3.360	−11.740	13.496
	MSE	1.851	12.744	3.817 × 10^−2^
	PRD	147.232	386.353	21.145
	NCC	0.0647	0.0862	0.9804

**Table 2:** Performance metrics achieved by the ISS method and two comparative denoising methods for measured vibration signals with different excitation frequencies.

Excitation Frequency (Hz)	Performance Metric	SS	MBSS	ISS
800	ASNR (dB)	−51.878	−67.107	−9.303
	MSE	3.061 × 10^−4^	1.020 × 10^−2^	1.692 × 10^−8^
	PRD	3.926 × 10^4^	2.267 × 10^5^	2.918 × 10^2^
	NCC	0.0081	0.0112	0.3242
850	ASNR (dB)	−51.709	−66.937	−14.566
	MSE	3.056 × 10^−4^	1.019 × 10^−2^	5.902 × 10^−8^
	PRD	3.850 × 10^4^	2.223 × 10^5^	5.350 × 10^2^
	NCC	0.0086	0.0114	0.1838
900	ASNR (dB)	−54.056	−69.285	−16.629
	MSE	3.055 × 10^−4^	1.018 × 10^−2^	5.524 × 10^−8^
	PRD	5.044 × 10^4^	2.912 × 10^5^	6.783 × 10^2^
	NCC	0.0060	0.0088	0.1458
950	ASNR (dB)	−52.080	−67.308	−13.934
	MSE	3.051 × 10^−4^	1.017 × 10^−2^	4.675 × 10^−8^
	PRD	4.018 × 10^4^	2.320 × 10^5^	4.974 × 10^2^
	NCC	0.0077	0.0113	0.1971
1000	ASNR (dB)	−49.556	−64.785	−9.885
	MSE	3.051 × 10^−4^	1.017 × 10^−2^	3.292 × 10^−8^
	PRD	3.005 × 10^4^	1.735 × 10^5^	3.121 × 10^2^
	NCC	0.0112	0.0107	0.3051
1050	ASNR (dB)	−55.348	−70.577	−16.374
	MSE	3.050 × 10^−4^	1.017 × 10^−2^	3.862 × 10^−8^
	PRD	5.853 × 10^4^	3.380 × 10^5^	6.587 × 10^2^
	NCC	0.0058	0.0053	0.1501

## Data Availability

The data presented in this study are available on request from the corresponding author. The data are not publicly available due to privacy and confidentiality agreements as well as other restrictions.

## References

[1] Hu Q., Si X.S., Zhang Q.H., Qin A.S. (2020). A Rotating Machinery Fault Diagnosis Method Based on Multi-Scale Dimensionless Indicators and Random Forests. Mech. Syst. Signal Process..

[2] Yan X., Liu Y., Jia M. (2020). Multiscale Cascading Deep Belief Network for Fault Identification of Rotating Machinery Under Various Working Conditions. Knowl.-Based Syst..

[3] Shen C., Qi Y., Wang J., Cai G., Zhu Z. (2018). An Automatic and Robust Features Learning Method for Rotating Machinery Fault Diagnosis Based on Contractive Autoencoder. Eng. Appl. Artif. Intell..

[4] Lu C., Wang Z.Y., Qin W.L., Ma J. (2017). Fault Diagnosis of Rotary Machinery Components Using a Stacked Denoising Autoencoder-Based Health State Identification. Signal Process..

[5] Qi H., Han D., Hou D., Wang C. (2023). A Novel Acoustic Emission Sensor Design and Modeling Method for Monitoring the Status of High-Speed Train Bearings. Struct. Health Monit..

[6] Knap P., Lalik K., Bałazy P. (2023). Boosted Convolutional Neural Network Algorithm for the Classification of the Bearing Fault Form 1-D Raw Sensor Data. Sensors.

[7] Tan X., Abu-Obeidah A., Bao Y., Nassif H., Nasreddine W. (2021). Measurement and Visualization of Strains and Cracks in CFRP Post-Tensioned Fiber Reinforced Concrete Beams Using Distributed Fiber Optic Sensors. Autom. Constr..

[8] Wijaya H., Rajeev P., Gad E. (2021). Distributed Optical Fibre Sensor for Infrastructure Monitoring: Field Applications. Opt. Fiber Technol..

[9] Badar M., Lu P., Wang Q., Boyer T., Chen K.P., Ohodnicki P.R. (2021). Real-Time Optical Fiber-Based Distributed Temperature Monitoring of Insulation Oil-Immersed Commercial Distribution Power Transformer. IEEE Sens. J..

[10] Fu C., Sui R., Peng Z., Meng Y., Zhong H., Shan R., Liang W., Liao C., Yin X., Wang Y. (2023). Wide-Range OFDR Strain Sensor Based on the Femtosecond-Laser-Inscribed Weak Fiber Bragg Grating Array. Opt. Lett..

[11] Zhang S., He J., Yu Q., Wu X. (2020). Multi-Scale Load Identification System Based on Distributed Optical Fiber and Local FBG-Based Vibration Sensors. Optik.

[12] Bachar L., Klein R., Tur M., Bortman J. (2022). Fault Diagnosis of Gear Transmissions Via Optic Fiber Bragg Grating Strain Sensors. Mech. Syst. Signal Process..

[13] Sánchez-Botello X., Roig R., Torre O.D.L., Madrigal J., Sales S., Escaler X. (2023). Assessment of Fiber Bragg Grating Sensors for Monitoring Shaft Vibrations of Hydraulic Turbines. Sensors.

[14] Vaddadi V.S.C.S., Parne S.R., Parambil V.V., Panda S.S.S., Gandi S. (2023). Design of Fiber Bragg Grating Sensor for Eccentricity Measurements in Ball Bearings. IEEE Trans. Instrum. Meas..

[15] Xu L., Yan Y. (2004). Wavelet-Based Removal of Sinusoidal Interference from A Signal. Meas. Sci. Technol..

[16] Yang H., Lin H., Ding K. (2018). Sliding Window Denoising K-Singular Value Decomposition and Its Application on Rolling Bearing Impact Fault Diagnosis. J. Sound Vib..

[17] Qi P., Gong S., Jiang N., Dai Y., Yang J., Jiang L., Tong J. (2023). Mattress-Based Non-Influencing Sleep Apnea Monitoring System. Sensors.

[18] Mao Q., Fang X., Hu Y., Li G. (2018). Chiller Sensor Fault Detection Based on Empirical Mode Decomposition Threshold Denoising and Principal Component Analysis. Appl. Therm. Eng..

[19] Hoseinzadeh M.S., Khadem S.E., Sadooghi M.S. (2018). Quantitative Diagnosis for Bearing Faults by Improving Ensemble Empirical Mode Decomposition. ISA Trans..

[20] Cheng X.R., Cui B.J., Hou S.Z. (2022). Fault Line Selection of Distribution Network Based on Modified CEEMDAN and Googlenet Neural Network. IEEE Sens. J..

[21] Wang Y., Markert R. (2016). Filter Bank Property of Variational Mode Decomposition and Its Applications. Signal Process..

[22] Patali P., Kassim S.T. (2021). High Throughput and Energy Efficient Linear Phase FIR Filter Architectures. Microprocess. Microsyst..

[23] Iqbal N., Zerguine A., Kaka S., Al-Shuhail A. (2018). Observation-Driven Method Based on IIR Wiener Filter for Microseismic Data Denoising. Pure Appl. Geophys..

[24] Chauchat P., Vilà-Valls J., Chaumette E. (2022). On the Asymptotic Behavior of Linearly Constrained Filters for Robust Multi-Channel Signal Processing. Signal Process..

[25] Mourad N., Reilly J.P., Kirubarajan T. (2017). Majorization-Minimization for Blind Source Separation of Sparse Sources. Signal Process..

[26] Zhang S., Zheng W.X., Zhang J.S. (2017). A New Combined-Step-Size Normalized Least Mean Square Algorithm for Cyclostationary Inputs. Signal Process..

[27] Boll S. (1979). Suppression of Acoustic Noise in Speech Using Spectral Subtraction. IEEE Trans. Acoust. Speech Signal Process..

[28] Alonso J.B., Cabrera J., Shyamnani R., Travieso C.M., Bolaños F., García A., Villegas A., Wainwright M. (2017). Automatic Anuran Identification Using Noise Removal and Audio Activity Detection. Expert Syst. Appl..

[29] Kompella K.C.D., Mannam V.G.R., Rayapudi S.R. (2018). Bearing Fault Detection in A 3 Phase Induction Motor Using Stator Current Frequency Spectral Subtraction with Various Wavelet Decomposition Techniques. Ain Shams Eng. J..

[30] Lockwood P., Boudy J. (1992). Experiments with A Nonlinear Spectral Subtractor (NSS), Hidden Markov Models and the Projection, for Robust Speech Recognition in Cars. Speech Commun..

[31] Dahlan R., Krisnandi D., Ramdan A., Pardede H.F. Unbiased Noise Estimator for Q-Spectral Subtraction Based Speech Enhancement. Proceedings of the 2019 International Conference on Radar, Antenna, Microwave, Electronics, and Telecommunications (ICRAMET).

[32] Kamath S.D., Loizou P.C. A Multi-Band Spectral Subtraction Method for Enhancing Speech Corrupted by Colored Noise. Proceedings of the 2002 IEEE International Conference on Acoustics, Speech and Signal Processing (ICASSP).

[33] Peeters C., Guillaume P., Helsen J. (2018). Vibration-Based Bearing Fault Detection for Operations and Maintenance Cost Reduction in Wind Energy. Renew. Energy.

[34] Peeters C., Guillaume P., Helsen J. (2017). A Comparison of Cepstral Editing Methods as Signal Pre-Processing Techniques for Vibration-Based Bearing Fault Detection. Mech. Syst. Signal Process..

[35] Tong Q., Huang L., Han H., He C. Multi-Band Spectral Subtraction Based on Adaptive Noise Estimation and Spectral Floor Optimization. Proceedings of the 2021 4th International Conference on Artificial Intelligence and Pattern Recognition (ICAIPR).

[36] Miyazaki R., Saruwatari H., Nakamura S., Shikano K., Kondo K., Blanchette J., Bouchard M. (2014). Musical-Noise-Free Blind Speech Extraction Integrating Microphone Array and Iterative Spectral Subtraction. Signal Process..

[37] Li C., Jiang T., Wu S. (2021). Single-Channel Speech Enhancement Based on Improved Frame-Iterative Spectral Subtraction in the Modulation Domain. China Commun..

[38] Xin L., Li Z., Gui X., Fu X., Fan M., Wang J., Wang H. (2020). Surface Intrusion Event Identification for Subway Tunnels Using Ultra-Weak FBG Array Based Fiber Sensing. Opt. Express.

[39] Ozawa K., Morise M., Sakamoto S., Watanabe K. Sound Source Separation by Spectral Subtraction Based on Instantaneous Estimation of Noise Spectrum. Proceedings of the 2019 6th International Conference on Systems and Informatics (ICSAI).

[40] Balaji V.R., Maheswaran S., Babu M.R., Kowsigan M., Prabhu E., Venkatachalam K. (2020). Combining Statistical Models Using Modified Spectral Subtraction Method for Embedded System. Microprocess. Microsyst..

[41] Chen S., Wang J., Zhang C., Li M., Li N., Wu H., Liu Y., Peng W., Song Y. (2023). Marine Structural Health Monitoring with Optical Fiber Sensors: A Review. Sensors.

[42] Fan Q., Jia Z.A., Feng D., Yong Z. (2021). Highly Sensitive FBG Pressure Sensor Based on Square Diaphragm. Optik.

[43] Guo Z., Gong X., Han J., Liu L., Wu Y., Meng F., Kang J. (2022). Research on a Multiscale Denoising Method for Low Signal-to-Noise Magnetotelluric Signal. IEEE Trans. Geosci. Remote Sens..

[44] Golmohammadi A., Hasheminejad N., Hernando D., Vanlanduit S., Bergh W.V.D. (2024). Performance Assessment of Discrete Wavelet Transform for De-Noising of FBG Sensors Signals Embedded in Asphalt Pavement. Opt. Fiber Technol..

